# The leverage role of feedback framing in promoting the spillover effect of idle item recycling

**DOI:** 10.3389/fpsyg.2025.1629839

**Published:** 2025-08-01

**Authors:** Tingting Guo, Jinlei Zhu, Zhengxiang Wu

**Affiliations:** ^1^College of Business Administration, Liaoning Technical University, Huludao, Liaoning, China; ^2^School of Business Administration, Dongbei University of Finance and Economics, Dalian, Liaoning, China

**Keywords:** spillover effect, idle item recycling, feedback framing, perceived impact, pride, goal progress

## Abstract

In response to the growing concerns over resource wastage and environmental pollution caused by improper disposal of idle items, information interventions have been implemented to encourage consumers to recycle these items. While such interventions primarily aim to promote idle item recycling, they may also trigger spillover effects either enhancing (positive spillover) or diminishing (negative spillover) additional environmental or non-environmental virtuous behaviors. This research investigates the impact of feedback framing on the spillover effects of consumers’ idle item recycling behavior through three scenario experiments. The results demonstrated that compared with highlighting the positive outcomes or benefits (positive framing), feedback information focused on preventing negative consequences or costs (negative framing) would strengthen the positive spillover of consumers’ idle item recycling behavior. And spillover effects are more likely to occur from idle item recycling to other low-cost pro-environmental behaviors. Furthermore, negative framing enhances consumers’ perceived impact and pride regarding their recycling actions, which subsequently amplifies the spillover effect. However, this effect is contingent on the level of goal progress; negative framing is effective only when goal progress is low, not when it is high. This study advances the theoretical understanding of feedback framing’s role in behavioral spillovers and offers practical insights for organizations and enterprises as they attempt to effectively intervene in consumers’ idle item recycling behavior.

## 1 Introduction

With the continuous improvement of consumers’ spending power and the growing popularity of e-commerce platforms, a lot of people have accumulated a large number of unused items, such as electronic products, clothing, furniture, etc., many of which are left untouched for years or simply discarded directly. For example, statistics of China National Material Recycling Association show that the number of idle mobile phones produced in China is as high as 600 million to 700 million every year, but few of them are recycled (China National Material Recycling Association, 2024). The waste problem caused by improper disposal of those idle items is becoming more and more serious, which not only leads to inefficient use of resources, but also causes multiple negative impacts on the environment and economy (Luo et al., 2019), as evidenced by notable incidents like the dead lakes in North America and the forest dieback in West Germany (Savari et al., 2023). Given the urgency of these problems, many municipalities in the world have provided incentive measures, formulated recycling policies and proposed recycling initiatives to reduce pollution of air, water, and other environmental resources caused by waste and discarded items, however, the overall recycling rate is still low (Reijonen et al., 2021). Currently, scholars are actively exploring ways to enhance consumer participation in recycling and have found that information interventions can be effective in promoting individuals’ recycling behavior (Varotto and Spagnolli, 2017; Luo et al., 2019; Wu et al., 2021; Wang et al., 2021; Lin and Nayga, 2022; Li et al., 2024). However, these researches have primarily focused on the recycling of waste materials. Unlike traditional recycling, which typically involves the disposal of everyday waste (e.g., plastic bottles) and is often perceived as a routine task driven by external regulations or infrastructure, idle-item recycling focuses on unused but still functional items, such as old furniture, clothing, or unused books. This form of recycling is more reliant on individual initiative and emphasizes the preservation of value rather than waste elimination (Li et al., 2019; Wu et al., 2021). As a result, it may evoke stronger moral motivations and a heightened sense of social responsibility, foster deeper psychological engagement, ultimately elicit distinct spillover effects.

Existing research has shown that a specific behavior in a certain field can enhance or inhibit other behaviors in the field; in other words, there is a spillover effect between behaviors (Susewind and Hoelzl, 2014; Behn et al., 2025). Spillover effects can be both positive and negative. Positive spillover is an effect that a behavior enhances the possibility of individuals implementing related behavior or the same behavior at different times and environments. Alternatively, if the implementation of a specific behavior reduces the probability of individuals implementing related or the same behavior at different times and environments, a negative spillover effect occurs (Truelove et al., 2014; Carrico, 2021). In recent years, scholars have placed significant emphasis on pro-environmental behavior spillover, yet studies have reported contradictory findings (Dolan and Galizzi, 2015; Geiger et al., 2021; Behn et al., 2025). Some studies have indicated that adopting one kind of pro-environmental behavior can have positive effects on other pro-environmental behaviors (Truelove et al., 2014; Lacasse, 2016; Nilsson et al., 2017; Carrico et al., 2018; Geng et al., 2019; Truelove and Nugent 2020; Bösehans et al., 2020; Ye et al., 2022; Taghvaee et al., 2023), while others have shown that engaging in one kind of pro-environmental behavior can have detrimental effects on other environmental (Lanzini and Thøgersen, 2014; Meijers et al., 2019) or non-environmental virtuous behaviors (Susewind and Hoelzl, 2014; Burger et al., 2022). However, some studies failed to find any spillover (Wolstenholme et al., 2020). In terms of the spillover of recycling, studies have also shown different results. For example, the results of Kidwell et al. (2013) indicated that consumers’ waste recycling behavior increases their willingness to buy environment-friendly products; but the research results of Truelove et al. (2016) demonstrated that the recycling behavior of democrats would lower their environmental identities and then less likely to support green fund. Importantly, the propensity for positive or negative spillover effects stemming from consumers’ pro-environmental behaviors appears to be contingent upon contextual factors (e.g., situational cues), and the strategic provision of information can serve as an effective mechanism to foster such spillover effects (Dolan and Galizzi, 2015; Nash et al., 2017; Carrico et al., 2018; Carfora et al., 2019; Carlsson et al., 2021; Ling et al., 2023). For instance, Geng et al. (2019) and Evans et al. (2013) found that both environmental and monetary framing are effective in encouraging electricity-saving behavior, but emphasizing environmental benefits is more likely to cause positive spillover, whereas monetary framing may trigger negative spillover. Tiefenbeck et al. (2013) found that feedback residents about their water consumption lowered their water use but increased their energy consumption. However, Carlsson et al. (2021) found that water conservation campaigns not only reduce water usage but also decrease electricity consumption.

Studies have proved that information interventions are effective in promoting spillover effect, however, the role of information interventions specifically targeting idle item recycling in inducing behavioral spillover effects remains underexplored. Given distinctions between traditional waste recycling and idle item recycling, it is essential to examine how informational interventions shape the spillover effects of idle item recycling on other behaviors, as well as to explore the underlying psychological mechanisms that mediate these effects. Therefore, rather than focusing on the direct impact of individuals’ recycling behavior on other behaviors, the goal of this study is to focus on ways to energize individuals’ idle item recycling actions to generate multiplier effects. Specifically, it conducts a comprehensive examination of whether differently framed feedback can elicit differentiated spillover effects and the underlying psychological mechanisms driving these effects. The main contribution of this study is to offer new insights into the pivotal role of message framing in driving behavioral spillover within the context of idle item recycling. From a managerial perspective, these findings provide practical guidance on leveraging interventions in recycling programs to foster meaningful pro-environmental behaviors among consumers.

## 2 Literature review

### 2.1 Behavioral spillover effects

The spillover effect refers to the phenomenon that the adoption of a first behavior can raise (positive spillover) or reduce (negative spillover) individuals to engage in the same behavior at a different time (i.e., spillover across time) or other behaviors in related fields (i.e., spillover across behaviors) or other contexts (i.e., spillover across contexts) (Littleford et al., 2014; Susewind and Hoelzl, 2014; Truelove et al., 2014; Dolan and Galizzi, 2015; Truelove et al., 2016; Nilsson et al., 2017; Taghvaee et al., 2023; Behn et al., 2025). Positive spillovers are likely to be driven by behavioral consistency and self-identity effect (Geiger et al., 2021). The consistency suggests that individuals have a strong motivation for behavioral consistency and tend to act in a way that is consistent with their previous behavior (Gawronski and Strack, 2012; Lalot et al., 2018). In other words, individuals are likely to exhibit more environmentally friendly behaviors in their subsequent actions after engaging in one pro-environmental behavior (Gawronski and Strack, 2012; Lanzini and Thøgersen, 2014; Thøgersen and Noblet, 2012). Self-identity refers to the way individuals label and describe themselves (Whitmarsh and O’Neill, 2010). Individuals who act environmentally friendly behaviors in the past may lead them to see themselves as “environmentalists” or “green,” and this will directly strengthen their environmental self-identity, then increases the possibility of performing other pro-environmental behaviors (Van der Werff et al., 2013, 2014a, 2014b; Lacasse, 2016; Elf et al., 2019; Meijers et al., 2019; Truelove and Nugent, 2020; Carrico, 2021; Khan, 2024). These findings suggest that amplifying individuals’ pro-environmental actions by reinforcing their pro-environmental identity and behavioral labeling. Negative spillovers are likely to be caused by moral licensing (Maki et al., 2019; Burger et al., 2022). The moral licensing effect refers to the fact that initial morally virtuous behavior improves individuals’ moral self-image, which in turn licenses individuals to engage in anti-social or immoral behavior (Merritt et al., 2010; Blanken et al., 2015; Nash et al., 2017). Studies in the field of pro-environmental behavior have indicated that the performance of an initial pro-environmental behavior can license individuals to behave badly in subsequent environmental behavior (Klöckner et al., 2013; Tiefenbeck et al., 2013; Clot et al., 2016; Geng et al., 2016; Lalot et al., 2018; Maki et al., 2019; Meijers et al., 2019) or other prosocial behavior (Susewind and Hoelzl, 2014).

To promote positive spillover in consumers’ pro-environmental behaviors, scholars have explored the effectiveness of information interventions as a means to stimulate such effects (Dogan et al., 2014; Truelove et al., 2014; Nilsson et al., 2017; Carlsson et al., 2021; Thøgersen et al., 2024). Framing interventions around environmental benefits, financial savings, and health advantages are the most common approaches to encourage individuals to engage in more pro-environmental behaviors (Van der Werff et al., 2014b; Maki et al., 2019; Meijers et al., 2019; Behn et al., 2025). For example, Lanzini and Thøgersen (2014) suggested that a stronger positive behavioral spillover effect of monetary inducement from purchasing green products on other environmentally friendly behaviors than verbal encouragement and praise. Dogan et al. (2014) emphasized that participants under environmental feedback showed a higher tendency of eco-driving behavior than those under financial feedback. Meanwhile, Steinhorst et al. (2015) found that the feedback framing of environmental and monetary both enhances individuals’ intentions to save electricity, but the positive spillover on climate-friendly intentions was only found in the environmental framing condition. Shreedhar and Galizzi (2021) found that a combined personal and planetary health framing of vegetarian diets increased charitable donations and short-term vegetarian food choices. In general, these studies focus on exploring the differences and impacts of these interventions in promoting the spillover effect of pro-environmental behavior. However, Existing studies have overlooked a key issue: the framing of a specific aspect in different ways can yield distinct effects. Moreover, existing studies have predominantly examined the outcomes of information strategies, with limited attention to the underlying psychological mechanisms driving these effects.

### 2.2 Message framing in environmental contexts

Message framing is a strategic form of information communication, it elicits differential responses to identical information content through variations in presentation and wording (Levin et al., 1998). This strategy operates by aligning information with the motivational underpinnings of specific behavior, thereby enhancing the salience and persuasiveness of corresponding beliefs. These reinforced beliefs subsequently influence individuals’ cognitive framework, shaping their attention toward particular benefits or costs associated with a given behavior. Message framing can be either positive or negative, positive framing emphasizes the positive consequences/benefits of performing promoted behavior, while negative framing highlights the negative outcomes/costs of not performing promoted behavior (Zhang et al., 2018; Yoon et al., 2019; Florence et al., 2022). Previous studies on message framing have yielded mixed findings regarding its effectiveness in promoting pro-environmental behaviors. While some studies highlight the superiority of positive framing in eliciting sustainable actions (Kim and Kim, 2014; Yoon et al., 2019; Chi et al., 2021), others underscore the greater impact of negative framing due to its stronger emotional impact (Grazzini et al., 2018; Gier et al., 2023). Nonetheless, some scholars contend that single message frames alone do not reliably enhance sustainable consumer behavior, and suggest that a combination of multiple frames may be more effective (Florence et al., 2022). Recent research further reveals that both positive and negative frames are effective, though positive framing yields stronger outcomes, whereas neutral messages show limited influence (Li et al., 2024). However, studies on the role of message framing in mitigating or amplifying positive spillover effects of consumers’ recycling behavior remain limited.

## 3 Hypotheses development

### 3.1 The effect of feedback framing on spillover

According to the theory of message framing, feedback on consumers’ idle item recycling behavior can be structured in two ways: positive framing, which accentuates the favorable results and advantages of recycling, and negative framing, which draws attention to the detrimental effects or losses that can be mitigated through recycling. Given that recycling behavior does not yield immediate and tangible benefits to recyclers, consumers often exhibit ambivalent and complex attitudes toward idle item recycling. However, when strategically designed feedback framing effectively conveys the societal and environmental value generated through recycling, it has the potential to reshape consumers’ perception of value associated with prosocial and ethical behaviors, thereby triggering a behavioral spillover effect that promotes participation in a broader range of sustainable and virtuous practices. Since negative framing evokes consumers’ perceptions of responsibility, pain, grief and shame (Liberman et al., 2005; Amatulli et al., 2017), negative framed information may be more effective than positive ones in promoting green or other virtuous behaviors (Bullard and Manchanda, 2013; Homar and Cvelbar, 2021). For example, negative framed (vs. positive framed) messages are more effective in persuading consumers to adopt recycling (Blose et al., 2015; Grazzini et al., 2018; Poortinga and Whitaker, 2018) and green consumption behaviors (Amatulli et al., 2017). The fundamental purpose of calling for idle item recycling is to protect the environment and avoid wasting resources. If negative framing is more effective in persuading consumers’ other prosocial or pro-environmental behaviors, we therefore speculate that compared with positive framing, when the feedback strategy focuses on avoiding negative outcomes, it conveys a sense of urgency to recyclers, stimulates their environmental values and beliefs, and enhances their environmental self-identity (Lin and Nayga, 2022), which in turn works better in persuading consumers to engage in more environmental protection behavior (Khan, 2024). By contrast, the positive framing makes consumers feel that their idle item recycling behavior has made a “best contribution” to the pro-environmental programs, which fails to build their environmental self-identity, and even reduces the possibility to practice other environmental protection behaviors because of the emergence of “moral licensing effect.” Therefore, we hypothesize that:

*H1:* Compared with positive framing, negative framing generates a stronger behavioral spillover effect on consumers’ idle item recycling behavior.

### 3.2 The mediating role of perceived impact

Perceived impact refers to individuals’ perception of the outcome of their behavior; it can also be referred to as perceived utility or perceived efficacy (Erlandsson et al., 2015; Wu et al., 2021). It reflects individual’s cognitive assessment of whether their actions can produce meaningful outcomes, and is a key prerequisite for individual pro-environmental action (Hornsey et al., 2021). Bullard and Penner (2017) argued that philanthropic giving involves impacting others’ lives and giving up one’s resources. Having an impact on others’ lives represents the reason for supporting philanthropy while giving up one’s resources represents how this occurs. Contributing to the well-being of others is desirable, while giving up one’s resources is undesirable; perceived impact is the critical factor that promotes one to give up resources and engage in virtuous behaviors (Erlandsson et al., 2015; Bullard and Penner, 2017). Cryder et al. (2013) stated that the greater the impact that individuals perceive their prosocial behavior, the more likely they perform prosocial behaviors. That is, the perceived impact was positively correlated with individuals’ prosocial behaviors. Meanwhile, Scholars have found that the information type affects consumers’ perceived impact of their behavior, and this perception in turn affects their behavioral intention or behavior (Hornsey et al., 2021). Combined with previous discussion, we argue that if negative framing feedback of idle item recycling behavior can easily activate clues related to virtuous behavior in consumers’ minds, then it can reasonably convince consumers that their individual idle item recycling action can make a difference. The greater the difference consumers believe their idle item recycling behavior can have, the more likely they are to perform other socially conscious behaviors. Therefore, we hypothesize that:

*H2: N*egative framing yields a greater perceived impact of idle item recycling compared to positive framing, and perceived impact mediates the relationship between negative framing and the spillover effect.

### 3.3 The mediating role of pride

Organizations often rely on positive emotions (e.g., pride, love and compassion) to encourage charitable donation, pro-environmental buying, and recycling. The underlying assumption seems to be that if consumers feel good, they are more likely to do so. Existing studies have shown that positive emotions have a profound influence on regulating consumers’ prosocial, moral and pro-environmental behaviors (Sun and Trudel, 2017; Liang et al., 2019; Christner et al., 2020). As a common example of self-conscious emotions, pride is a positive self-conscious emotion arising from the attainment of achievements or the realization of values (Shipley and van Riper, 2022; Jiao et al., 2023). And it motivates prosocial behaviors by reinforcing one’s sense of having made a difference (Antonetti and Maklan, 2014). For example, Manika et al. (2021) suggested that pride induced by environmentally-friendly technology adoption increases the likelihood of engaging in conservation behaviors. Message framing systematically influences consumers’ decision-making through dual processing pathways, the cognitive route (rational cognition) and the affective route (emotional arousal) (Gier et al., 2023). when the feedback message of idle item recycling behavior is framed as negative, it conveys that the behavior avoids negative outcomes, and invokes consumers’ positive emotions of pride associated with such behavior (Sun and Trudel, 2017). Moreover, the feeling of pride reinforces consumers’ environmental self-identity (Ma et al., 2019), then encourage them to perform more socially desired behaviors in the future. This means the greater the pride consumers feel after acting idle item recycling behavior, the more likely they are to engage in future virtuous actions. Therefore, we hypothesize that:

*H3:* Pride will mediate the relationship between negative framing and the spillover effect.

**Figure 1:**
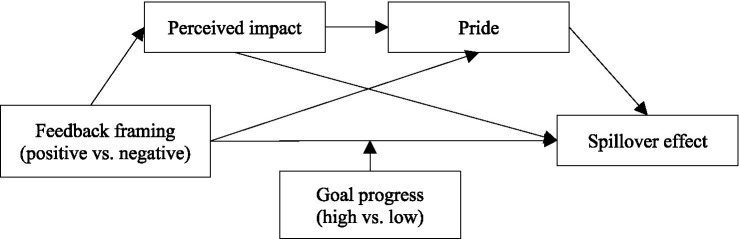
Conceptual framework.

As described above, negative framing feedback can not only influence the spillover effect by enhancing consumers’ perception of the impact of their behavior, but also by arousing consumers’ pride. An important question is, what is the relationship between perceived impact and pride in the process of negative framing feedback driving spillover effect? According to the Cognitive-Affective Personality System (CAPS) theory (Mischel and Shoda, 1995), situational stimuli activate contextual encoding processes, triggering situation-appropriate cognitive appraisals and affective responses, ultimately generating a dynamic “situation-cognition-affection-behavior” feedback loop. Therefore, this study believes that the stronger consumers perceive the impact of their own idle item recycling behavior, the more likely they believe their behavior will achieve specific achievements, and this perception stimulates their sense of pride, thus enhancing their likelihood of performing other virtuous behaviors in the future. In other words, the impact of negative framing on the spillover effect may have a path of “negative framing-perceived impact-pride-spillover effect” (as shown in Figure 1). Therefore, we hypothesize that:

*H4:* There is a chain mediation effect of perceived impact and pride between negative framing and the spillover effect.

### 3.4 The moderating role of goal progress

The goal process refers to the progression toward an abstract, desired state (Fishbach and Dhar, 2005). Although goal pursuit is inherently subjective, existing research suggests that individuals’ perceptions of goal attainability and progress are significantly shaped by objective indicators of goal progress. That is, objective goal progress not only reflects the actual degree of task completion, but also influences individuals’ psychological assessments of feasibility, remaining effort, and motivational engagement (Fishbach and Dhar, 2005; Fishbach et al., 2006; Bonezzi et al., 2011). In practice, enterprises or social organizations often provide consumers with real-time feedback on the progress or outcome of idle item recycling to encourage sustained engagement. Such progress feedback informs consumers of the gap between what has been accomplished and what remains to be done, which in turn affects consumers’ perception of the accessibility and feasibility of the goal and the efforts needed to achieve it (Koo and Fishbach, 2014). According to Park and Hedgcock (2016), consumers regulate their behaviors by assessing the discrepancy between the current state and the reference state. A high level of goal progress implies proximity to the end and less effort is needed, whereas low level of goal progress signals insufficient effort and a greater need for action.

Idle item recycling activity with higher goal progress signals that the recycling goal is accessibility, and the behavior is identified by group members. Therefore, consumers are more susceptible to high goal progress rather than low progress (Ku et al., 2018). Positive framing feedback emphasizes the benefit or positive outcome of idle item recycling, it gives consumers a perception of social recognition, which in turn enhances their motivation and commitment to idle item recycling goals and prompts them to work hard to achieve recycling goals. Simultaneously, it heightens their tendency to engage in other similar or related behaviors, as inconsistent behaviors trigger negative self-evaluative emotions that consumers naturally wish to reduce these negative feelings (Tuk et al., 2021). Conversely, idle item recycling activity with lower goal progress induces a sense that the recycling goal lacks progress. This may lead consumers to perceive the goal as both unattainable and lacking public recognition, making them indifferent to the objectives. Negative framing, which posits the avoidable losses of recycling idle items, can be more persuasive than positive framing due to loss aversion-people’s tendency to prefer avoiding losses over acquiring equivalent gains (Levin et al., 1998). Negative framing triggers a heightened sense of responsibility and obligation, making consumers more likely to take corrective action (Van der Werff et al., 2014a). This, in turn, enhances consumers’ perceived impact of recycling behavior, thereby encouraging the adoption of broader virtuous behaviors. Therefore, we hypothesize that:

*H5:* Relative to negative framing, positive framing will lead to a stronger spillover effect when the goal process is high; however, when goal progress is low, negative framing will be more effective in enhancing the spillover effect.

Drawing upon the above literature and analysis, we developed the conceptual framework, as shown in Figure 1.

## 4 Methodology and hypotheses test

### 4.1 Prior research

The purpose of the pre-test was to select suitable experimental stimuli for this study. According to the statistics from 36kr.com and Zhiyan Consulting, top idle items on second-hand e-commerce platforms include books, handbags, clothing and shoes, as well as digital products like smartphones, laptops, and iPads. Books, handbags, clothing and shoes are experience products, while digital products are search products; consumers’ perceptions of these two types of products are different (Jiménez and Mendoza, 2013). To control for the potential impact of product type on the experimental results, both categories were included in subsequent experiments. Stimuli were chosen to ensure no significant differences in participants’ familiarity or perceived recycling suitability.

A total of 125 participants were invited to take part in the study, and the sample characteristics are shown in Table 1. Participants first read an informed consent form and confirmed their voluntary participation in the survey. They then completed a demographic questionnaire, followed by assessments of their familiarity (i.e., You are familiar with this kind of idle item) with each product and the perceived suitability of recycling each item (3 items, e.g., I think it is logical for this kind of idle item to be recycled, α = 0.863). Each item was assessed on a 7-point scale (1 = strongly disagree, 7 = totally agree).

**Table 1:** Sample characteristics of pilot study.

Characteristics	Options	Frequency	Percentage (%)
Gender	Male	64	51.20
	Female	61	48.80
Age	≤18	0	0.00
	19–25	32	25.60
	26–30	47	37.60
	31–35	25	20.00
	36–40	13	10.40
	41–45	3	2.40
	≥46	5	4.00
Occupation	Student	15	12.00
	Enterprise and institution staff	77	61.60
	Civil servant	23	18.40
	Farmer	4	3.20
	Others	6	4.80
Education	Below college degree	9	7.20
	College degree	40	32.00
	Bachelor’s degree	62	49.60
	Master’s degree and above	14	11.20

As shown in Table 2, the results revealed that participants were familiar with these six alternative products and their means of familiarity were greater than the median value of 4. Moreover, participants perceived that there was no difference between iPad and books as idle items to be recycled [*t*(248)_familarity_ = 0.929, *p* = 0.074, Cohen’s *d* = 0.258; *t*(248)_suitability_ = 0.142, *p* = 0.739, Cohen’s *d* = 0.039]. To ensure the scientific nature of the research results, iPad and books were selected as experimental stimulus items for the follow-up experiments.

**Table 2:** Evaluation form of experimental alternative products.

Source	Mean					
	Smartphone	iPad	Laptop	Books	Handbags	Clothing and shoes
Familiarity	5.444	5.037	4.815	5.260	5.148	4.630
Suitability	5.667	5.210	5.444	5.235	4.790	4.815

### 4.2 Study 1

In the first study, we aimed to investigate the impact of feedback framing of idle item recycling on participants’ attitudes toward related pro-environmental behaviors (e.g., green consumption, purchasing second-hand products, energy conservation, garbage classification recycling) and other non-environmental moral behaviors (e.g., voluntary activities, charitable donation, and blood donation) (Lanzini and Thøgersen, 2014). In addition, we included a control condition with no information regarding framing. We predicted that the spillover effect would be stronger in the negative framing condition than in the other two conditions (H1).

#### 4.2.1 Method

One hundred sixty-two university students (*M*_age_ = 22.06 years, 43.80% male) participated in exchange for course credit. They were randomly assigned to one of the three conditions (positive framing vs. negative framing vs. control) of a between-subjects design. As recommended by Khalil et al. (2021), each experimental condition collected approximately 50 participants. And the priori power analysis indicated that this sample is sufficient to detect a medium effect (*f* = 0.25) with an alpha level of 0.05 and a power of 80% (G*Power 3.1; Faul et al., 2007). This effect size is widely recognized as a standard benchmark of medium effect in experimental and behavioral science research, and has been extensively adopted in consumer behavior and marketing studies (Peterson, 2001). In Experiment 1, iPad was selected as the stimulus object. To avoid experimenter bias and the Hawthorne effect, researchers did not perform the experiment. Neither the experimental operators nor participants knew the purpose of the study. Before the experiment began, participants read an informed consent form and confirmed their voluntary participation. They were also informed that there were no right or wrong answers, and their participation was completely anonymous.

All participants were randomly assigned to one of three conditions in which feedback framing (positive vs. negative vs. control) was manipulated. As recalling past moral or immoral actions can affect individuals’ future moral behavior (Jordan et al., 2011), an experimental scenario was applied to study 1. Specifically, participants were introduced to read a scenario about an idle item recycling program in which an online recycling platform ELEG (a fictitious company was used to avoid any potential confounding effect) was initiated, and they recycled their idle iPad in accordance with the operation process. In the positive framing condition, the feedback message presented the potential benefits of recycling. In the negative framing condition, it emphasized the avoidance of the negative consequences of their recycling behavior. The control group received only neutral information. After reading the respective scenarios, two items (“To what extent did the feedback message focus on benefits/losses would be gained/avoided if people do recycle idle iPad”) were measured on a 7-point scale (1 = not at all, 7 = very much) (White et al., 2011) to identify the focus conveyed by the scenario.

Subsequently, they were asked to complete a series of questionnaire measures. First, they completed green consumption scale with the question “How likely that you would purchase green products in the future?” followed by adjective pairs on a 7-point scale including *likely/unlikely*, *possible/impossible*, *and probable/improbable* (Chang et al., 2015). Then, they reported their likelihood of participating in three non-pro-environmental moral activities (i.e., volunteering activity, charitable donation, blood donation) in the following month (Lanzini and Thøgersen, 2014), their willingness to buy second-hand products with four items (e.g., When necessary, I would consider buying second-hand product.) (Dodds et al., 1991), and their intention of energy conservation with five items (e.g., Printing documents on both sides.) (Lanzini and Thøgersen, 2014), and their intention to recycle household garbage separately over the next week with two items (e.g., I intend to recycle household garbage separately over the next week.) (Chu and Chiu, 2003). Finally, participants ostensibly read five unrelated questions, such that they were less likely to be aware of the actual purpose of the study. Each item was assessed on a 7-point scale (1 = very unlikely, 7 = very likely). The measures were combined via factor scores into the variables “green consumption” (*α* = 0.878), “energy conservation” (*α* = 0.891), “household garbage classification recycling” (*α* = 0.811), “non-pro-environmental moral behaviors” (*α* = 0.814), and “second-hand products purchasing intention” (*α* = 0.794).

In addition, participants answered two demographic questions (age and gender), and an open-ended question related to their perception of the experiment. Which revealed that age and gender were not significantly different across experimental conditions, and participants were not aware of the purpose of the research.

#### 4.2.2 Data analysis and results

The framing manipulation was successfully implemented, with participants in the positive framing condition perceiving that the information focused on improving positive outcomes of recycling idle iPads [*M*_positive_ = 5.123, *M*_negative_ = 3.907, *t*(106) = 6.722, *p* < 0.001, Cohen’s *d* = 1.306], whereas those in the negative framing condition perceived it focused on preventing the negative consequences [*M*_positive_ = 3.667, *M*_negative_ = 4.889, *t*(106) = 6.697, *p* < 0.001, Cohen’s *d* = 1.301].

We performed ANOVA on the spillover effect elicited by the framing effect. The results revealed the main effects of feedback framing on green consumption [*F*(2,159) = 16.799, *p* < 0.001, *η*^2^ = 0.174], energy conservation [*F*(2,159) = 13.818, *p* < 0.001, *η*^2^ = 0.148], household garbage classification recycling behavior [*F*(2,159) = 9.772, *p* < 0.001, *η*^2^ = 0.109]; but not on second-hand products purchasing intention [*F*(2,159) = 2.077, *p* = 0.129, *η*^2^ = 0.125], and other non-pro-environmental moral behaviors [*F*(2,159) = 1.667, *p* = 0.192, *η*^2^ = 0.021]. Then we further tested the effects of feedback framing on the spillover effect with multiple comparisons of Tukey HSD. As shown in Table 3, compared to positive framing and control, negative framing elicited a greater level of green consumption, energy conservation, and household garbage classification recycling behavior. Moreover, in comparison to the control condition, positive framing induced greater intention of green consumption, energy conservation, and household garbage classification recycling behavior. Two types of feedback framing have no obvious spillover effect on the second-hand products purchase intention and non-pro-environmental moral behaviors.

**Table 3:** Multiple comparisons of Tukey HSD.

Items	Feedback framing	Mean	SD	Groups	*p*
Green consumption	Positive framing	4.444	0.772	Positive framing vs. Negative framing	0.044
	Negative framing	4.753	0.677	Negative framing vs. Control	0.000
	Control	3.926	0.795	Positive framing vs. Control	0.001
Energy conservation	Positive framing	4.763	0.670	Positive framing vs. Negative framing	0.028
	Negative framing	5.096	0.636	Negative framing vs. Control	0.000
	Control	4.419	0.703	Positive framing vs. Control	0.023
Household garbage classification recycling	Positive framing	4.648	0.822	Positive framing vs. Negative framing	0.024
	Negative framing	4.963	0.643	Negative framing vs. Control	0.000
	Control	4.352	0.677	Positive framing vs. Control	0.034
Non-pro-environmental moral behaviors	Positive framing	4.593	0.571	Positive framing vs. Negative framing	0.822
	Negative framing	4.667	0.691	Negative framing vs. Control	0.175
	Control	4.444	0.664	Positive framing vs. Control	0.458
Second-hand products purchasing intention	Positive framing	3.468	0.581	Positive framing vs. Negative framing	0.112
	Negative framing	3.769	0.783	Negative framing vs. Control	0.414
	Control	3.579	0.925	Positive framing vs. Control	0.738

This study provides initial evidence of the framing effect on the spillover of consumers’ idle item recycling behavior. Specifically, compared with positive framing, feedback on idle item recycling behavior with a negative framework will enhance the diagnosis of this behavior, and motivate individuals to behave more pro-environmental behaviors. However, it is difficult to improve the spillover effect of idle item recycling behavior on non-pro-environmental moral behaviors and second-hand product purchasing intention. In general, the results of Study 1 confirmed our main effect conjecture that the feedback message with negative framing is more effective than positive framing in eliciting a positive spillover effect (H1). We will further investigate the underlying mechanisms in subsequent studies.

### 4.3 Study 2

Study 2 aimed to test the conjecture of H2, H3, and H4 that negative framing would yield greater perceived impact and pride, which in turn promotes the spillover effect of idle item recycling. In addition, it extends the results of Study 1 by testing the framing effect in a different product category.

#### 4.3.1 Method

A total of 150 adults were recruited to participate in the study, and they were randomly assigned to one of two conditions (positive framing vs. negative framing) in a between-subjects design. Seven questionnaires were eliminated, as participants completed less than 70% of the questionnaire items. The remaining 143 participants consisted of 45.50% male and 54.50% female participants; and the sample characteristics are shown in Table 4. Their ages were mainly ranged from 19 to 35; 57.34% were employees of enterprises or institutions, and most of the participants had a college education or higher (76.2%). The sample structure better represents the basic characteristics of individuals who are involved in idle item recycling in China. And the sample size was determined by *a priori* power analysis, which indicated that a minimum of *N* = 128 is sufficient to detect a medium effect (*f* = 0.25) with an alpha level of 0.05 and a power of 80% (G*Power 3.1; Faul et al., 2007).

**Table 4:** Sample characteristics of Study 2.

Characteristics	Options	Frequency	Percentage (%)
Gender	Male	65	45.50
	Female	78	54.50
Age	≤18	2	1.40
	19–25	39	27.27
	26–30	54	37.76
	31–35	26	18.18
	36–40	16	11.19
	41–45	4	2.80
	≥46	2	1.40
Occupation	Student	19	13.29
	Enterprise and institution staff	82	57.34
	Civil servant	17	11.89
	Farmer	29	13.98
	Others	5	3.50
Education	Below college degree	8	5.59
	College degree	26	18.18
	Bachelor’s degree	88	61.54
	Master’s degree and above	21	14.69

#### 4.3.2 Procedure

As in Study 1, participants first completed the informed consent process, then read a scenario about an idle item recycling program facilitated by an online platform. However, in this case, the program specifically focused on recycling idle books. Subsequently, participants were asked to complete the message framing manipulation items. They then filled out a series of questionnaire measures about their attitudes toward green consumption, energy conservation, household garbage classification recycling, non-pro-environmental moral behaviors, second-hand products purchasing intention, and five other unrelated questions. In addition, participants were also required to complete a perceived impact scale comprising three items (e.g., I believe that the expected consequences are very positive.) (Erlandsson et al., 2015; Lee et al., 2019; Wu et al., 2021) and a pride scale consisting of two items (e.g., I am proud of my idle book recycling efforts.) (Harth et al., 2013; Ma et al., 2019). All items were assessed on a 7-point Likert scale. The measures were combined via factor scores into the variables “green consumption” (*α* = 0.700), “energy conservation” (*α* = 0.776), “household garbage classification recycling” (*α* = 0.905), “non-pro-environmental moral behaviors” (*α* = 0.821), “second-hand products purchasing intention” (*α* = 0.875), “perceived impact” (*α* = 0.827), and “pride” (*α* = 0.770).

#### 4.3.3 Data analysis and results

First, the framing manipulation was tested, and the Independent-Samples *T*-test showed that participants in the positive framing condition indicated that the case was focused on improving positive outcomes [*M*_positive_ = 4.987, *M*_negatvie_ = 3.882, *t*(141) = 6.114, *p* < 0.001, Cohen’s *d* = 1.030]; and the negative framing condition focused on preventing negative consequences [*M*_positive_ = 3.947, *M*_negative_ = 5.059, *t*(141) = 7.605, *p* < 0.001, Cohen’s *d* = 1.280].

Subsequently, we conducted a one-way ANOVA with message framing as the independent variable, green consumption, energy conservation, household garbage classification recycling, non-pro-environmental moral behaviors, and second-hand products purchasing intention as dependent variables. The results were the same as in Study 1, participants in the negative framing condition reported higher intention of green consumption [*M*_positive_ = 4.698, *M*_negative_ = 4.936, *F*(1,141) = 6.205, *p* = 0.014, *η*^2^ = 0.128], energy conservation [*M*_positive_ = 5.019, *M*_negative_ = 5.218, *F*(1,141) = 4.644, *p* = 0.033, *η*^2^ = 0.032], household garbage classification recycling [*M*_positive_ = 4.847, *M*_negative_ = 5.559, *F*(1,141) = 20.777, *p* < 0.001, *η*^2^ = 0.128]; however, both positive framing and negative framing showed non-significant effects on participants’ intention to perform non-pro-environmental moral behaviors [*M*_positive_ = 4.520, *M*_negative_ = 4.417, *F*(1,141) = 0.519, *p* = 0.472, =0.004], and second-hand products purchasing intention [*M*_positive_ = 3.927, *M*_negative_ = 3.787, *F*(1,141) = 0.606, *p* = 0.438, *η*^2^ = 0.004]. The results confirm the findings of Study 1.

**Figure 2:**
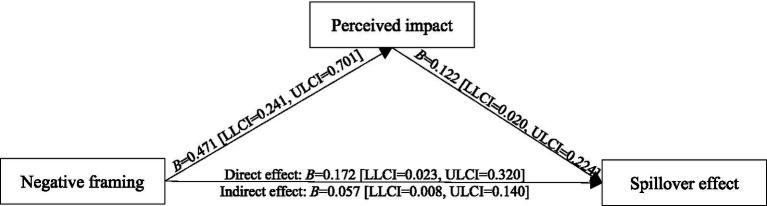
The mediating effect of perceived impact.

**Figure 3:**
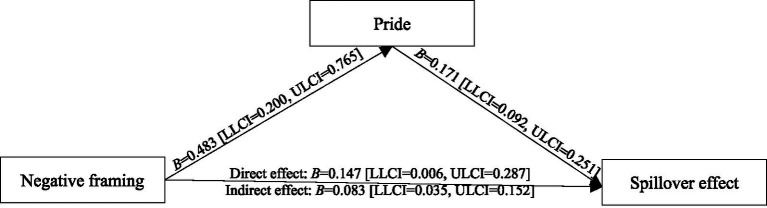
The mediating effect of pride.

Next, we performed one-way ANOVA to analyze the perceived impact and pride elicited by the framing effect, and the data analysis showed that the framing effect had significant effect on perceived impact [*M*_positive_ = 4.618, *M*_negative_ = 5.088, *F*(1,141) = 16.351, *p* < 0.001, *η*^2^ = 0.104] and pride [*M*_positive_ = 4.473, *M*_negative_ = 4.956, *F*(1,141) = 11.389, *p* < 0.001, *η*^2^ = 0.075]. The results indicated that, compared with positive framing, negative framing triggers participants to perceive their idle item recycling behavior to be more impactful and elicits more feelings of pride. Accordingly, the mediation effects of perceived impact and pride were assessed with Hayes (2013, model 4) SPSS PROCESS Macro. Using the bootstrapping technique, the model analysis results revealed that feedback framing had a direct effect on perceived impact (*B* = 0.471, *SE* = 0.116, *t* = 4.044, 95% confidence interval [LLCI = 0.241, ULCI = 0.701], *p* < 0.001), which in turn significantly affected the spillover effect (*B* = 0.122, *SE* = 0.052, *t* = 2.370, 95% confidence interval [LLCI = 0.020, ULCI = 0.224], *p =* 0.019). More importantly, the indirect effect value of perceived impact between feedback framing and spillover effect was significant (*B* = 0.057, *SE* = 0.033, 95% confidence interval [LLCI = 0.008, ULCI = 0.140]). As shown in Figure 2. The results indicated that perceived impact mediated the effect of negative framing on the spillover effect. Thus, H2 is supported. Meanwhile, the model analysis results revealed that feedback framing had a direct effect on pride (*B* = 0.483, *SE* = 0.143, *t* = 3.375, 95% confidence interval [LLCI = 0.200, ULCI = 0.765], *p* < 0.001), which in turn significantly affected the spillover effect (*B* = 0.171, *SE* = 0.040, *t* = 4.262, 95% confidence interval [LLCI = 0.092, ULCI = 0.251], *p* < 0.001), and the indirect effect value of pride between feedback framing on the spillover effect was significant (*B* = 0.083, *SE* = 0.030, 95% confidence interval [LLCI = 0.035, ULCI = 0.152]). As shown in Figure 3. The results showed that pride mediated the impact of negative framing on the spillover effect and validated our conjecture of H3.

Furthermore, the value of the chain mediating effects of perceived impact and pride was assessed with Hayes (2013, model 6) SPSS PROCESS Macro. As predicted in H4, the indirect effect of feedback framing on spillover effect was mediated by a perceived impact → pride pathway (*B* = 0.018, *SE* = 0.012, 95% confidence interval [LLCI = 0.003, ULCI = 0.052]). The results of the chain mediation analysis are shown in the Table 5, and results supported H4.

**Table 5:** Results of the chain mediation analysis.

Paths	Effect (*B*)	Confidence intervals (95%)
Feedback framing → perceived impact → spillover effect	0.040	[−0.006, 0.109]
Feedback framing → pride → spillover effect	0.059	[0.017, 0.120]
Feedback framing → perceived impact → pride → spillover effect	0.018	[0.003, 0.052]

Study 2 replicated the findings of study 1. Meanwhile, study 2 investigated the mediating role of perceived impact and pride. Supported by theoretical derivation and data analysis, this study confirms the mechanism underlying the effect of negative framing on the spillover effect. That is, negative framing, which focuses on preventing or avoiding negative outcomes, is perceived to be more impactful and easier to elicit feelings of pride, which in turn increases the total spillover effect. It is noteworthy that negative framing enhances the perception of impact, which would predict pride and subsequently drive the spillover effect.

### 4.4 Study 3

The purpose of Study 3 was to test our proposition that positive framing would be more effective in enhancing the spillover effect than negative framing when goal progress is high. By contrast, negative framing is more likely to elicit a spillover effect when goal progress is low.

#### 4.4.1 Method

Study 3 was a 2 (feedback framing: positive vs. negative) × 2 (goal progress: high vs. low) between-subjects design. Two hundred participants were randomly assigned to one of the four conditions, but 31 had to be dropped from the analysis because they did not complete the survey properly. Thus, 169 valid questionnaires were obtained and further analyzed. *A priori* power analysis indicates that this sample is sufficient to detect a medium effect (*f* = 0.25) with an alpha level of 0.05, and a power of 90% (G*Power 3.1; Faul et al., 2007). The descriptive statistics of the sample are shown in Table 6.

**Table 6:** Sample characteristics of Study 3.

Characteristics	Options	Frequency	Percentage (%)
Gender	Male	81	47.93
	Female	88	52.07
Age	≤18	2	1.18
	19–25	40	23.67
	26–30	67	39.64
	31–35	34	20.12
	36–40	16	9.47
	41–45	9	5.33
	46 and above	1	0.59
Occupation	Student	26	15.38
	Enterprise and institution staff	97	57.40
	Civil servant	10	5.92
	Farmer	35	20.71
	Others	1	0.59
Education	Below college degree	9	5.33
	College degree	47	27.81
	Bachelor’s degree	95	56.21
	Master’s degree and above	18	10.65

#### 4.4.2 Procedure

Following the experimental design of related research (Jordan et al., 2011; Park and Hedgcock, 2016; Steinhorst et al., 2015), participants were asked to read a real research report titled *Rural Children’s Reading Report*, which was jointly issued by the China Foundation for Poverty Alleviation and Beijing Normal University, after completing the informed consent process. Feedback framing and goal progress were manipulated at the end of the report. The feedback framing was manipulated by stating the positive (vs. negative) consequences if those children had (vs. did not have) enough books to read, and the contribution made by their idle books recycling behavior. Following prior studies of Park and Hedgcock (2016), goal progress was manipulated by informing participants that the idle item recycling platform ELEG had launched a project to collect 10,000 books for children in underprivileged rural areas of Central China. Participants were told the project had reached either 70% (high progress) or 30% (low progress) of its goal. After reading this report, participants were asked to evaluate the information type with two measurement questions as in study 1, and indicate their perceived goal progress on a 7-point scale (1 = no progress; 7 = a lot of progress). Subsequently, participants filled out a questionnaire about their attitudes toward green consumption (*α* = 0.858), energy conservation (*α* = 0.849), and household garbage classification recycling (*α* = 0.830) as in study 2. To avoid the purpose of the research being guessed, participants were told that several unrelated investigations would be combined to reduce costs and improve the efficiency of the investigation.

#### 4.4.3 Data analysis and results

First, an Independent-Sample *T*-test was conducted to check whether the manipulations of message framing and goal progress were successful. As expected, the results showed that participants in the positive framing condition reported that the report focused more on the positive consequences of recycling idle books [*M*_positive_ = 5.094, *M*_negative_ = 4.357, *t*(167) = 4.768, *p* < 0.001, Cohen’s *d* = 0.738], whereas the negative framing condition focused on negative consequences that the idle books recycling behavior prevented [*M*_positive_ = 4.400, *M*_negative_ = 5.095, *t*(167) = 5.169, *p* < 0.001, Cohen’s *d* = 0.800]. Additionally, participants in the high progress condition perceived greater progress than those in the low progress [*M*_positive_ = 4.915, *M*_negative_ = 3.402, *t*(167) = 9.378, *p* < 0.001, Cohen’s *d* = 1.451].

**Figure 4:**
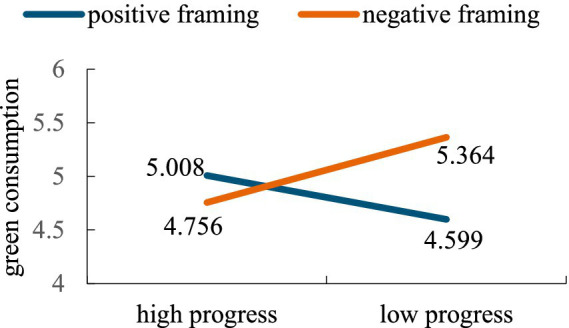
The interaction effect of feedback framing and goal progress on green consumption.

The main effects of feedback framing, goal progress and their interaction on green consumption were estimated by two-way ANOVA. The results revealed that the main effect of feedback framing was significant [*F*(1,165) = 4.025, *p* = 0.046, *η*^2^ = 0.022], and the main effect of goal progress was non-significant [*F*(1,165) = 0.601, *p* = 0.439, *η*^2^ = 0.003]. More importantly, the interaction between feedback framing and goal progress was significant [*F*(1,165) = 15.7972, *p* < 0.001, *η*^2^ = 0.085]. Further comparative analysis revealed that in the high progress condition, neither positive nor negative framing significantly influenced green consumption [*M*_positive_ = 5.008, *M*_negative_ = 4.756, *F*(1,166) = 1.89, *p* = 0.172]. However, in the case of low goal progress, the negative framing was more effective than positive framing [*M*_positive_ = 4.599, *M*_negative_ = 5.364, *F*(1,166) = 18.42, *p* < 0.001]. As shown in Figure 4.

**Figure 5:**
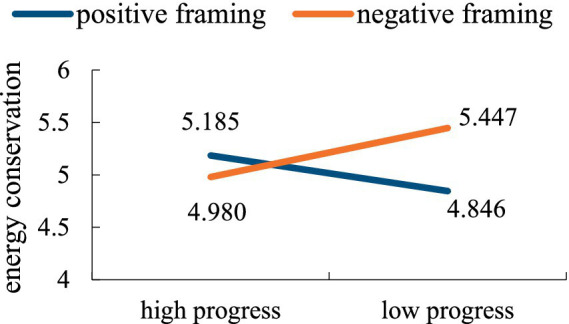
The interaction effect of feedback framing and goal progress on energy conservation.

With energy conservation as the dependent variable, the results demonstrated a significant main effect of feedback framing [*F*(1,165) = 4.772, *p* = 0.030, *η*^2^ = 0.025] and an interaction between feedback framing and goal progress [*F*(1,165) = 19.747, *p* < 0.001, *η*^2^ = 0.104], the main effect of goal progress was not significant [*F*(1,165) = 0.484, *p* = 0.482, *η*^2^ = 0.003]. Further comparative analysis showed that the framing effect had no significant effect on energy conservation in the case of high goal progress [*M*_positive_ = 5.185, *M*_negative_ = 4.980, *F*(1,166) = 2.49, *p* = 0.117]. Negative framing was more effective than positive framing when the goal progress was low [*M*_positive_ = 4.846, *M*_negative_ = 5.447, *F*(1,166) = 22.65, *p* < 0.001]. As shown in Figure 5.

**Figure 6:**
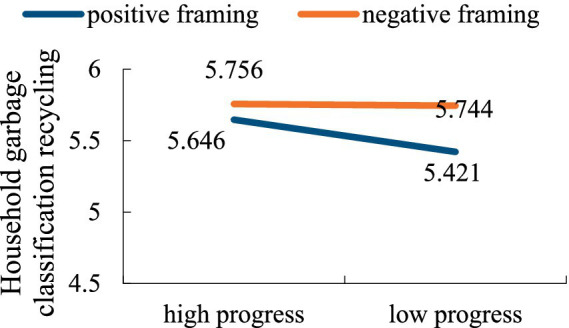
The interaction effect of feedback framing and goal progress on household garbage classification recycling.

Subsequently, the effects of feedback framing, goal progress and their interaction on household garbage classification recycling were tested. The results confirmed that the effect of feedback framing was significant [*F*(1,165) = 4.176, *p* = 0.043, *η*^2^ = 0.024], however, the main effect of goal progress [*F*(1,165) = 1.257, *p* = 0.264, *η*^2^ = 0.007] was non-significant, and the interaction between feedback framing and goal progress was marginally significant [*F*(1,165) = 2.018, *p* = 0.071, *η*^2^ = 0.055]. Although the interaction effect on household garbage classification recycling is only marginally significant, the results still reveal a potential interaction between message framing and goal progress in influencing consumers’ household garbage classification recycling behavior, suggesting that further analysis is warranted. Further comparative analysis indicated that the framing effect had a non-significant effect on household garbage recycling intention when the goal progress was high [*M*_positive_ = 5.646, *M*_negative_ = 5.756, *F*(1,166) = 0.52, *p* = 0.472]. Negative framing was more effective than positive framing when the goal progress was low [*M*_positive_ = 5.421, *M*_negative_ = 5.744, *F*(1,166) = 4.83, *p* = 0.029]. As shown in Figure 6.

**Figure 7:**
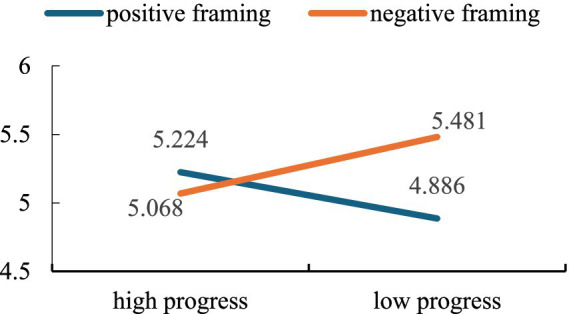
Moderating effect of goal progress on the relationship between feedback framing and spillover effect.

Finally, with the mean of the variables of green consumption, energy conservation and household garbage recycling intention as dependent variable, the two-way ANOVA revealed that the main effect of feedback framing was significant [*M*_positive_ = 5.049, *M*_negative_ = 5.280, *F*(1,165) = 11.599, *p* < 0.001, *η*^2^ = 0.055], however, the main effect of goal progress [*M*_high_ = 5.146, *M*_low_ = 5.181, *F*(1,165) = 0.339, *p* = 0.561, *η*^2^ = 0.002] was non-significant, and the interaction between feedback framing and goal progress was significant [*F*(1,165) = 33.967, *p* < 0.001, *η*^2^ = 0.160]. Specifically, neither positive nor negative framing significantly influenced the spillover effect when the goal progress was high [*M*_positive_ = 5.224, *M*_negative_ = 5.068, *F*(1,166) = 2.86, *p* = 0.093]. However, in the case of low goal progress, negative framing was more effective than positive framing [*M*_positive_ = 4.886, *M*_negative_ = 5.481, *F*(1,166) = 23.45, *p* < 0.001]. As shown in Figure 7, hypothesis H5 was partially supported.

The results of this study again confirmed the findings of Study 1, that is, negative framing was more likely to elicit the spillover effect of idle item recycling. Additionally, goal progress was tested as a potential boundary condition of the framing effect in this study. We found that while goal progress was not strong enough to alter the spillover effect of idle item recycling, it did influence the impact of the framing effect. Specifically, under the condition of high goal progress, neither positive nor negative framing significantly affected the spillover effect of idle item recycling. However, at low levels of goal progress, negative framing that focuses on preventing negative outcomes was more likely to facilitate the generalization of idle item recycling to other behaviors.

## 5 Conclusion and general discussion

### 5.1 General discussion

This study demonstrates the fundamental differences between idle item recycling and traditional waste recycling in psychological mechanisms and behavioral drivers. It further examines how feedback framing, as a key contextual cue, functions as an intervention strategy to trigger spillover effects from idle item recycling to other low-cost sustainable behaviors. In line with consistency theory findings, our results corroborated that feedback to consumers about their idle item recycling behavior increased their motivation to engage in other pro-environmental behaviors that are not costly (e.g., green consumption), but not in other virtuous behaviors with high cost (e.g., blood donation). In other words, positive spillover of idle item recycling behavior is more likely to occur in the domain of environmental protection and limited to activities that are easy and less costly to carry out (Thøgersen and Crompton, 2009; Lanzini and Thøgersen, 2014).

What is more, consumers are more likely to show behavioral consistency when a feedback message of one’s idle item recycling behavior is framed either negatively or positively, although the strength of these framing strategies differs. Specifically, consumers tend to engage in additional pro-environmental behaviors when recalling their idle item recycling behavior with prevention-focused lens (as demonstrated in Study 1 and Study 2) rather than promotion-focused ones. However, this result is observed only when the level of goal progress is low. When goal progress is high, the persuasion effectiveness between positive and negative framing does not differ (Study 3). These findings align with the goal progress commitment model (Fishbach and Dhar, 2005; Fishbach et al., 2006), which posits that individuals may activate either a commitment or a licensing pathway after perceiving progress toward a goal. Specifically, under low goal progress, negative feedback is more persuasive than positive feedback, likely because it highlights goal insufficiency and activates the commitment pathway, enhancing environmental responsibility and behavioral consistency. In contrast, high goal progress mitigates the framing effect, consistent with a licensing response. Moreover, as stated in hypotheses H2, H3 and H4, we revealed that perceived impact and pride were potential explanations for the relationship between negative framing and positive spillover (Study 2). Specifically, feedback on consumers’ idle item recycling behavior with negative framing was more likely to elicit consumers’ perception of their idle item recycling efforts had a meaningful impact on others, thereby fostering a sense of pride in their actions. This heightened perception of impact and pride, in turn, motivated consumers to engage in additional pro-environmental behaviors. These findings highlight the differential influence of message framing on consumers’ self-perceptions and subsequent behavioral outcomes, emphasizing the importance of strategic communication in promoting sustained pro-environmental engagement.

### 5.2 Theoretical implications

This article makes several theoretical contributions. First, while prior research has extensively examined factors influencing individuals’ recycling behavior and explored the spillover effects of such behavior. However, the focus of these studies has predominantly been on waste or garbage recycling rather than idle item recycling. This distinction is particularly important, as idle item recycling involves different psychological and behavioral mechanisms compared to traditional waste recycling. By elucidating these differences, unlike the study done by Sun et al. (2024), our findings advance theoretical understanding of recycling behavior and provide novel insights into how message framing strategies can be strategically employed to foster positive spillover effects in recycling across diverse contexts. Second, research has yielded contradictory conclusions regarding the spillover effects of recycling behavior. Some researchers have found that consumers are more likely to act in pro-environmental behaviors after recycling (Thøgersen and Noblet, 2012). This is because previous pro-environmental behaviors reinforce one’s self-identity as “environmentalists” or “green,” thereby promoting behaviors that align with that identity (Truelove and Nugent, 2020; Carrico, 2021; Khan, 2024). In contrast, studies based on the theory of moral licensing have shown that consumers are more likely to behave in less environmentally behaviors, such as increased resource consumption, after recycling (Nilsson et al., 2017; Sun and Trudel, 2017; Ma et al., 2019). Our findings extend existing research by demonstrating that the spillover effect of consumers’ recycling behaviors for idle items is more likely to be influenced by information intervention, and tend to occur in other low-cost pro-environmental behaviors. This suggests that informational intervention plays an important role in eliciting positive spillover, and that exploring spillover effects across different categories is essential. Third, this study confirms that individuals’ interpretation of goal progress feedback plays a critical role in influencing subsequent behavior. When goal progress is relatively high, it tends to diminish the impact of feedback framing. In contrast, when progress is relatively low, it is more likely to activate the commitment pathway and promote sustained pro-environmental behavior. These findings not only support the core assumptions of the goal progress commitment model but also provide important insights for designing effective green behavior interventions. Finally, prior studies have overlooked the internal psychological mechanisms underlying such spillover effect. Few studies have examined the mediating role of negative emotions (e.g., guilt) in spillover effects (Grazzini et al., 2018; Liang et al., 2019; Burger et al., 2022). These studies suggest that information interventions that evoke negative emotions in consumers can prompt them to engage in pro-environmental behaviors in order to alleviate discomfort. However, negative emotions (e.g., guilt) typically focus on identifying problems and initiating corrective actions (Bissing-Olson et al., 2016). If consumers do not perceive their current behavior as contributing to environmental issues, or if they find justifications for their existing practices, they may be less inclined to engage in pro-environmental behaviors (Liang et al., 2019). Therefore, relying solely on the activation of negative emotions is unlikely to drive lasting changes in consumer behavior. Positive emotions, such as pride, not only reflect the emotional experience of favorable outcomes but also involve self-awareness that one’s pro-environmental behaviors align with personal goals and standards (Williams and Desteno, 2008). This emotional state encourages consumers to persist in efforts they believe are congruent with their personal objectives, making pride a stronger motivator of pro-environmental behavior (Antonetti and Maklan, 2014). This study uncovers the psychological mechanism driving the spillover effect in idle item recycling behavior, structured as ‘negative framing → perceived impact → pride → behavioral spillover,’ and elucidates the underlying psychological processes. The findings provide a novel theoretical perspective for understanding the mechanisms underlying environmental behavior motivation.

### 5.3 Management implications

Government agencies and recycling enterprises often allocate substantial resources to encourage consumer participation in idle item recycling through strategies such as monetary incentives and promotional campaigns. However, these efforts frequently yield limited results and may even prove counterproductive. Our research findings offer a novel perspective for social organizations and enterprises, suggesting that informational feedback mechanisms can serve as a more effective approach to guiding consumers to engage in more impactful pro-environmental behaviors. Specifically, the post-event informational feedback strategy with negative framing which highlights the losses and negative consequences prevented by one’s idle item recycling behavior, can lead consumers to perceive their recycling behavior has a greater impact on others, society or the environment. This heightened perception, in turn, reinforces consumers’ perception of pride, then enhances the likelihood of consumers performing other pro-environmental behaviors. Therefore, marketers should emphasize the negative consequences or the losses to be avoided and prevented by consumers’ idle item recycling behavior. For example, the core message of the feedback framework can be expressed as follows: By recycling your old mobile phone today, you have successfully prevented approximately 6,000 liters of soil from being contaminated by heavy metals. While recycling 5 kilograms of old clothing saved over 10,000 liters of water, equivalent to the water used for about 70 showers. These actions contribute significantly to a green, low-carbon lifestyle. However, such messaging should align with consumers’ perceived goal progress, which shapes their interpretation of feedback framing.

### 5.4 Limitation and future research direction

Although this study provides meaningful implications for scholars and practitioners, it also has some limitations. First, the information framework strategy explored in this study focuses on the behavior of idle item recycling. Future studies might benefit from exploring how to design effective information feedback strategies to integrate idle item recycling with other pro-environmental behaviors, therefore increase the correlation between behaviors, then enhance the effects of spillover effects. Second, although participants may exhibit strong intentions toward green consumption, energy conservation, or household waste recycling under the influence of feedback framing, these intentions do not necessarily translate into actual behavior due to factors such as limited convenience or time constraints. There may be a gap between actual action and intention (Hassan et al., 2016). To improve the validity of research results, future research might benefit from using more objective methods to measure these behaviors, such as measuring actual behavior over a certain period. Third, the data in this study are all from China. Future research could further validate the generalizability of the findings by collecting data from diverse countries or regions. Additionally, the present study did not explore whether perceived goal progress exerts spillover effects through its influence on perceived impact and pride. Future research could construct more sophisticated mediation models to examine this mechanism in greater depth.

## Data Availability

The raw data supporting the conclusions of this article will be made available by the authors, without undue reservation.

## References

[ref1] Amatulli C. De Angelis M. Peluso A. M. Soscia I. Guido G. (2017). The effect of negative message framing on green consumption: an investigation of the role of shame. J. Bus. Ethics 157, 605–615. doi: 10.1007/s10551-017-3677-1, PMID: 40693173

[ref2] Antonetti P. Maklan S. (2014). Feelings that make a difference: how guilt and pride convince consumers of the effectiveness of sustainable consumption choices. J. Bus. Ethics 124, 117–134. doi: 10.1007/s10551-013-1841-9

[ref3] Behn O. Wichmann J. Leyer M. Schilling A. (2025). Spillover effects in environmental behaviors: a scoping review about its antecedents, behaviors, and consequences. Curr. Psychol. 44, 3665–3689. doi: 10.1007/s12144-025-07431-9, PMID: 40693173

[ref4] Bissing-Olson M. J. Fielding K. S. Iyer A. (2016). Experiences of pride, not guilt, predict pro-environmental behavior when pro-environmental descriptive norms are more positive. J. Environ. Psychol. 45, 145–153. doi: 10.1016/j.jenvp.2016.01.001

[ref5] Blanken I. Van de Ven N. Zeelenberg M. (2015). A meta-analytic review of moral licensing. Personal. Soc. Psychol. Bull. 41, 540–558. doi: 10.1177/0146167215572134, PMID: 25716992

[ref6] Blose J. E. Mack R. W. Pitts R. E. (2015). The influence of message framing on hotel guests’ linen-reuse intentions. Cornell Hosp. Q. 56, 145–154. doi: 10.1177/1938965514556440

[ref7] Bonezzi A. Brendl C. M. De Angelis M. (2011). Stuck in the middle: the psychophysics of goal pursuit. Psychol. Sci. 22, 607–612. doi: 10.1177/0956797611404899, PMID: 21474842

[ref8] Bösehans G. Bolderdijk J. W. Wan J. (2020). Pay more, fly more? Examining the potential guilt-reducing and flight-encouraging effect of an integrated carbon offset. J. Environ. Psychol. 71:101469. doi: 10.1016/j.jenvp.2020.101469

[ref9] Bullard O. Manchanda R. V. (2013). Do sustainable products make us prevention focused? Mark. Lett. 24, 177–189. doi: 10.1007/s11002-012-9221-2

[ref10] Bullard O. Penner S. (2017). A regulatory-focused perspective on philanthropy: promotion focus motivates giving to prevention-framed causes. J. Bus. Res. 79, 173–180. doi: 10.1016/j.jbusres.2017.06.013

[ref11] Burger A. M. Schuler J. Eberling E. (2022). Guilty pleasures: moral licensing in climate-related behavior. Glob. Environ. Change 72:102415. doi: 10.1016/j.gloenvcha.2021.102415

[ref12] Carfora V. Catellani P. Caso D. Conner M. (2019). How to reduce red and processed meat consumption by daily text messages targeting environment or health benefits. J. Environ. Psychol. 65:101319. doi: 10.1016/j.jenvp.2019.101319

[ref13] Carlsson F. Jaime M. Villegas C. (2021). Behavioral spillover effects from a social information campaign. J. Environ. Econ. Manag. 109:102325. doi: 10.1016/j.jeem.2020.102325, PMID: 40690924

[ref14] Carrico A. R. (2021). Climate change, behavior, and the possibility of spillover effects: recent advances and future directions. Curr. Opin. Behav. Sci. 42, 76–82. doi: 10.1016/j.cobeha.2021.03.025

[ref15] Carrico A. R. Raimi K. T. Truelove H. B. Eby B. (2018). Putting your money where your mouth is: an experimental test of pro-environmental spillover from reducing meat consumption to monetary donations. Environ. Behav. 50, 723–748. doi: 10.1177/0013916517713067

[ref16] Chang H. Zhang L. Xie G. X. (2015). Message framing in green advertising: the effect of construal level and consumer environmental concern. Int. J. Advert. 34, 158–176. doi: 10.1080/02650487.2014.994731

[ref17] Chi O. H. Denton G. Gursoy D. (2021). Interactive effects of message framing and information content on carbon offsetting behaviors. Tour. Manag. 83:104244. doi: 10.1016/j.tourman.2020.104244, PMID: 33100457 PMC7574723

[ref9001] China National Material Recycling Association. (2024). Recycling: Turning old phones from waste to treasure. Retrieved from https://www.gov.cn/yaowen/liebiao/202406/content_6955247.htm

[ref18] Christner N. Pletti C. Paulus M. (2020). Emotion understanding and the moral self-concept as motivators of prosocial behavior in middle childhood. Cogn. Dev. 55:100893. doi: 10.1016/j.cogdev.2020.100893

[ref19] Chu P. Y. Chiu J. F. (2003). Factors influencing household waste recycling behavior: test of an integrated model. J. Appl. Soc. Psychol. 33, 604–626. doi: 10.1111/j.1559-1816.2003.tb01915.x

[ref20] Clot S. Grolleau G. Ibanez L. (2016). Do good deeds make bad people? Eur. J. Law Econ. 42, 491–513. doi: 10.1007/s10657-014-9441-4

[ref21] Cryder C. E. Loewenstein G. Scheines R. (2013). The donor is in the details. Organ. Behav. Hum. Decis. Process. 120, 15–23. doi: 10.1016/j.jesp.2013.07.003

[ref22] Dodds W. B. Monroe K. B. Grewal D. (1991). Effects of price, brand, and store information on buyers’ product evaluations. J. Mark. Res. 28, 307–319. doi: 10.1177/0022243791028003

[ref23] Dogan E. Bolderdijk J. W. Steg L. (2014). Making small numbers count: environmental and financial feedback in promoting eco-driving behaviors. J. Consum. Policy 37, 413–422. doi: 10.1007/s10603-014-9259-z

[ref24] Dolan P. Galizzi M. M. (2015). Like ripples on a pond: behavioral spillovers and their implications for research and policy. J. Econ. Psychol. 47, 1–16. doi: 10.1016/j.joep.2014.12.003

[ref25] Elf P. Gatersleben B. Christie I. (2019). Facilitating positive spillover effects: new insights from a mixed-methods approach exploring factors enabling people to live more sustainable lifestyles. Front. Psychol. 9:2699. doi: 10.3389/fpsyg.2018.02699, PMID: 30804866 PMC6371024

[ref26] Erlandsson A. Björklund F. Bäckström M. (2015). Emotional reactions, perceived impact and perceived responsibility mediate the identifiable victim effect, proportion dominance effect and in-group effect respectively. Organ. Behav. Hum. Decis. Process. 127, 1–14. doi: 10.1016/j.obhdp.2014.11.003

[ref27] Evans L. Maio G. R. Corner A. Hodgetts C. J. Ahmed S. Hahn U. (2013). Self-interest and pro-environmental behaviour. Nat. Clim. Chang. 3, 122–125. doi: 10.1038/nclimate1662

[ref28] Faul F. Erdfelder E. Lang A. G. Buchner A. (2007). G* power 3: a flexible statistical power analysis program for the social, behavioral, and biomedical sciences. Behav. Res. Methods 39, 175–191. doi: 10.3758/BF03193146 17695343

[ref29] Fishbach A. Dhar R. (2005). Goals as excuses or guides: the liberating effect of perceived goal progress on choice. J. Consum. Res. 32, 370–377. doi: 10.1086/497548

[ref30] Fishbach A. Dhar R. Zhang Y. (2006). Subgoals as substitutes or complements: the role of goal accessibility. J. Pers. Soc. Psychol. 91, 232–242. doi: 10.1037/0022-3514.91.2.232, PMID: 16881761

[ref31] Florence E. S. Fleischman D. Mulcahy R. (2022). Message framing effects on sustainable consumer behaviour: a systematic review and future research directions for social marketing. J. Soc. Mark. 12, 623–652. doi: 10.1108/JSOCM-09-2021-0221others

[ref32] Gawronski B. Strack F. (2012). Cognitive consistency: a fundamental principle in social cognition. New York, NY: Guilford Press.

[ref33] Geiger S. J. Brick C. Nalborczyk L. Bosshard A. Jostmann N. B. (2021). More green than gray? Toward a sustainable overview of environmental spillover effects: a Bayesian meta-analysis. J. Environ. Psychol. 78:101694. doi: 10.1016/j.jenvp.2021.101694

[ref34] Geng L. Chen Y. Ye L. Zhou K. Chen Y. (2019). How to predict future pro-environmental intention? The spillover effect of electricity-saving behavior under environmental and monetary framing. J. Clean. Prod. 233, 1029–1037. doi: 10.1016/j.jclepro.2019.06.088

[ref35] Geng L. Cheng X. Tang Z. Zhou K. Ye L. (2016). Can previous pro-environmental behaviours influence subsequent environmental behaviours? The licensing effect of pro-environmental behaviours. J. Pac. Rim Psychol. 10, 1–9. doi: 10.1017/prp.2016.6

[ref36] Gier N. R. Krampe C. Kenning P. (2023). Why it is good to communicate the bad: understanding the influence of message framing in persuasive communication on consumer decision-making processes. Front. Hum. Neurosci. 17:1085810. doi: 10.3389/fnhum.2023.1085810, PMID: 37731668 PMC10508293

[ref37] Grazzini L. Rodrigo P. Aiello G. Viglia G. (2018). Loss or gain? The role of message framing in hotel guests’ recycling behaviour. J. Sustain. Tour. 26, 1944–1966. doi: 10.1080/09669582.2018.1526294

[ref38] Harth N. S. Leach C. W. Kessler T. (2013). Guilt, anger, and pride about in-group environmental behaviour: different emotions predict distinct intentions. J. Environ. Psychol. 34, 18–26. doi: 10.1016/j.jenvp.2012.12.005

[ref39] Hassan L. M. Shiu E. Shaw D. (2016). Who says there is an intention–behaviour gap? Assessing the empirical evidence of an intention–behaviour gap in ethical consumption. J. Bus. Ethics 136, 219–236. doi: 10.1007/s10551-014-2440-0

[ref40] Hayes A. F. (2013). Introduction to mediation, moderation, and conditional process analysis: A regression-based approach. New York, NY: Guilford Press.

[ref41] Homar A. R. Cvelbar L. K. (2021). The effects of framing on environmental decisions: a systematic literature review. Ecol. Econ. 183:106950. doi: 10.1016/j.ecolecon.2021.106950

[ref42] Hornsey M. J. Chapman C. M. Oelrichs D. M. (2021). Ripple effects: can information about the collective impact of individual actions boost perceived efficacy about climate change? J. Exp. Soc. Psychol. 97:104217. doi: 10.1016/j.jesp.2021.104217

[ref43] Jiao L. Ren Z. Guo Z. Gao S. Xu Y. (2023). Individual differences in place attachment and pro-environmental behavior: pride as an emotional tie. Pers. Individ. Differ. 214:112357. doi: 10.1016/j.paid.2023.112357

[ref44] Jiménez F. R. Mendoza N. A. (2013). Too popular to ignore: the influence of online reviews on purchase intentions of search and experience products. J. Interact. Mark. 27, 226–235. doi: 10.1016/j.intmar.2013.04.001

[ref45] Jordan J. Mullen E. Murnighan J. K. (2011). Striving for the moral self: the effects of recalling past moral actions on future moral behavior. Personal. Soc. Psychol. Bull. 37, 701–713. doi: 10.1177/0146167211400208, PMID: 21402752

[ref46] Khalil M. Septianto F. Lang B. Northey G. (2021). The interactive effect of numerical precision and message framing in increasing consumer awareness of food waste issues. J. Retail. Consum. Serv. 60:102470. doi: 10.1016/j.jretconser.2021.102470

[ref47] Khan A. N. (2024). Elucidating the effects of environmental consciousness and environmental attitude on green travel behavior: moderating role of green self-efficacy. Sustain. Dev. 32, 2223–2232. doi: 10.1002/sd.2771

[ref48] Kidwell B. Farmer A. Hardesty D. M. (2013). Getting liberals and conservatives to go green: political ideology and congruent appeals. J. Consum. Res. 40, 350–367. doi: 10.1086/670610

[ref49] Kim S. B. Kim D. Y. (2014). The effects of message framing and source credibility on green messages in hotels. Cornell Hosp. Q. 55, 64–75. doi: 10.1177/1938965513503400

[ref50] Klöckner C. A. Nayum A. Mehmetoglu M. (2013). Positive and negative spillover effects from electric car purchase to car use. Transp. Res. Part D Transp. Environ. 21, 32–38. doi: 10.1016/j.trd.2013.02.007

[ref51] Koo M. Fishbach A. (2014). Dynamics of self-regulation: how (un)accomplished goal actions affect motivation. Motiv. Sci. 1, 73–90. doi: 10.1037/mot0000001 18211171

[ref52] Ku H. H. Yang P. H. Chang C. L. (2018). Reminding customers to be loyal: does message framing matter? Eur. J. Mark. 52, 783–810. doi: 10.1108/EJM-09-2016-0516

[ref53] Lacasse K. (2016). Don’t be satisfied, identify! Strengthening positive spillover by connecting pro-environmental behaviors to an “environmentalist” label. J. Environ. Psychol. 48, 149–158. doi: 10.1016/j.jenvp.2016.09.006

[ref54] Lalot F. Falomir-Pichastor J. M. Quiamzade A. (2018). Compensation and consistency effects in pro-environmental behaviour: the moderating role of majority and minority support for pro-environmental values. Group Process. Intergroup Relat. 21, 403–421. doi: 10.1177/1368430217733117

[ref55] Lanzini P. Thøgersen J. (2014). Behavioural spillover in the environmental domain: an intervention study. J. Environ. Psychol. 40, 381–390. doi: 10.1016/j.jenvp.2014.09.006

[ref56] Lee Y. J. Haley E. Yang K. (2019). The role of organizational perception, perceived consumer effectiveness and self-efficacy in recycling advocacy advertising effectiveness. Environ. Commun. 13, 239–254. doi: 10.1080/17524032.2017.1308407

[ref57] Levin I. P. Schneider S. L. Gaeth G. J. (1998). All frames are not created equal: a typology and critical analysis of framing effects. Organ. Behav. Hum. Decis. Process. 76, 149–188. doi: 10.1006/obhd.1998.2804, PMID: 9831520

[ref58] Li B. K. Guo T. T. Wu Z. X. (2019). Consumer’s participation intention of recycling idle items: from the perspective of self-construal. Chin. J. Manag 16, 736–746. doi: 10.3969/j.issn.1672-884x.2019.05.013

[ref59] Li Q. Zhai Q. Wang J. (2024). The impact of information intervention on urban residents’ willingness to sort domestic waste. J. Environ. Manag. 371:123201. doi: 10.1016/j.jenvman.2024.123201, PMID: 39509982

[ref60] Liang D. Hou C. Jo M. S. Sarigöllü E. (2019). Pollution avoidance and green purchase: the role of moral emotions. J. Clean. Prod. 210, 1301–1310. doi: 10.1016/j.jclepro.2018.11.103

[ref61] Liberman N. Idson L. C. Higgins E. T. (2005). Predicting the intensity of losses vs. non-gains and non-losses vs. gains in judging fairness and value: a test of the loss aversion explanation. J. Exp. Soc. Psychol. 41, 527–534. doi: 10.1016/j.jesp.2004.06.007

[ref62] Lin W. Nayga R. M. Jr. (2022). Green identity labeling, environmental information, and pro-environmental food choices. Food Policy 106:102187. doi: 10.1016/j.foodpol.2021.102187

[ref63] Ling M. Xu L. Yang H. (2023). Direct and spillover effects of social norm nudges for household recycling: a longitudinal field experiment. Sustain. Prod. Consum. 42, 423–433. doi: 10.1016/j.spc.2023.06.001

[ref64] Littleford C. Ryley T. J. Firth S. K. (2014). Context, control and the spillover of energy use behaviours between office and home settings. J. Environ. Psychol. 40, 157–166. doi: 10.1016/j.jenvp.2014.06.002

[ref65] Luo Y. Zelenika I. Zhao J. (2019). Providing immediate feedback improves recycling and composting accuracy. J. Environ. Manag. 232, 445–454. doi: 10.1016/j.jenvman.2018.11.061, PMID: 30502613

[ref66] Ma B. Li X. Jiang Z. Jiang J. (2019). Recycle more, waste more? When recycling efforts increase resource consumption. J. Clean. Prod. 206, 870–877. doi: 10.1016/j.jclepro.2018.09.063

[ref67] Maki A. Carrico A. R. Raimi K. T. Truelove H. B. Araujo B. Yeung K. L. (2019). Meta-analysis of pro-environmental behaviour spillover. Nat. Sustain. 2, 307–315. doi: 10.1038/s41893-019-0263-9

[ref68] Manika D. Antonetti P. Papagiannidis S. Guo X. (2021). How pride triggered by pro-environmental technology adoption spills over into conservation behaviours: a social business application. Technol. Forecast. Soc. Change 172:121005. doi: 10.1016/j.techfore.2021.121005

[ref69] Meijers M. H. Noordewier M. K. Verlegh P. W. Willems W. Smit E. G. (2019). Paradoxical side effects of green advertising: how purchasing green products may instigate licensing effects for consumers with a weak environmental identity. Int. J. Advert. 38, 1202–1223. doi: 10.1080/02650487.2019.1607450

[ref70] Merritt A. C. Effron D. A. Monin B. (2010). Moral self-licensing: when being good frees us to be bad. Soc. Personal. Psychol. Compass 4, 344–357. doi: 10.1111/j.1751-9004.2010.00263.x

[ref71] Mischel W. Shoda Y. (1995). A cognitive-affective system theory of personality: Reconceptualizing situations, dispositions, dynamics, and invariance in personality structure. Psychol. Rev. 102, 246–268. doi: 10.1037/0033-295X.102.2.246, PMID: 7740090

[ref72] Nash N. Whitmarsh L. Capstick S. Hargreaves T. Poortinga W. Thomas G.. (2017). Climate-relevant behavioral spillover and the potential contribution of social practice theory. Wiley Interdiscip. Rev. Clim. Chang. 8:e481. doi: 10.1002/wcc.481

[ref73] Nilsson A. Bergquist M. Schultz W. P. (2017). Spillover effects in environmental behaviors, across time and context: a review and research agenda. Environ. Educ. Res. 23, 573–589. doi: 10.1080/13504622.2016.1250148

[ref74] Park J. Hedgcock W. M. (2016). Thinking concretely or abstractly: the influence of fit between goal progress and goal construal on subsequent self-regulation. J. Consum. Psychol. 26, 395–409. doi: 10.1016/j.jcps.2015.12.003

[ref75] Peterson R. A. (2001). On the use of college students in social science research: insights from a second-order meta-analysis. J. Consum. Res. 28, 450–461. doi: 10.1086/323732

[ref76] Poortinga W. Whitaker L. (2018). Promoting the use of reusable coffee cups through environmental messaging, the provision of alternatives and financial incentives. Sustainability 10:873. doi: 10.3390/su10030873

[ref77] Reijonen H. Bellman S. Murphy J. Kokkonen H. (2021). Factors related to recycling plastic packaging in Finland’s new waste management scheme. Waste Manag. 131, 88–97. doi: 10.1016/j.wasman.2021.05.034, PMID: 34111827

[ref78] Savari M. Sheheytavi A. Amghani M. S. (2023). Factors underpinning Iranian farmers' intention to conserve biodiversity at the farm level. J. Nat. Conserv. 73:126419. doi: 10.1016/j.jnc.2023.126419

[ref79] Shipley N. J. van Riper C. J. (2022). Pride and guilt predict pro-environmental behavior: a meta-analysis of correlational and experimental evidence. J. Environ. Psychol. 79:101753. doi: 10.1016/j.jenvp.2021.101753

[ref80] Shreedhar G. Galizzi M. M. (2021). Personal or planetary health? Direct, spillover and carryover effects of non-monetary benefits of vegetarian behaviour. J. Environ. Psychol. 78:101710. doi: 10.1016/j.jenvp.2021.101710

[ref81] Steinhorst J. Klöckner C. A. Matthies E. (2015). Saving electricity–for the money or the environment? Risks of limiting pro-environmental spillover when using monetary framing. J. Environ. Psychol. 43, 125–135. doi: 10.1016/j.jenvp.2015.05.005

[ref82] Sun N. Liu D. Zhang J. (2024). Exploring the factors influencing the intention to clothing and textiles recycling among Chinese college students’: a study based on TPB and VBN. Front. Psychol. 14:1328037. doi: 10.3389/fpsyg.2023.1328037, PMID: 38274694 PMC10808640

[ref83] Sun M. Trudel R. (2017). The effect of recycling versus trashing on consumption: theory and experimental evidence. J. Mark. Res. 54, 293–305. doi: 10.1509/jmr.15.0574

[ref84] Susewind M. Hoelzl E. (2014). A matter of perspective: why past moral behavior can sometimes encourage and other times discourage future moral striving. J. Appl. Soc. Psychol. 44, 201–209. doi: 10.1111/jasp.12214

[ref85] Taghvaee V. M. Nodehi M. Arani A. A. Jafari Y. Shirazi J. K. (2023). Sustainability spillover effects of social, environment and economy: mapping global sustainable development in a systematic analysis. Asia Pac. J. Reg. Sci. 7, 329–353. doi: 10.1007/s41685-022-00231-0

[ref86] Thøgersen J. Crompton T. (2009). Simple and painless? The limitations of spillover in environmental campaigning. J. Consum. Policy 32, 141–163. doi: 10.1007/s10603-009-9101-1

[ref87] Thøgersen J. Noblet C. (2012). Does green consumerism increase the acceptance of wind power? Energy Policy 51, 854–862. doi: 10.1016/j.enpol.2012.09.044

[ref88] Thøgersen J. Vatn A. Aasen M. (2024). The chicken or the egg? Spillover between private climate action and climate policy support. J. Environ. Psychol. 99:102434. doi: 10.1016/j.jenvp.2024.102434

[ref89] Tiefenbeck V. Staake T. Roth K. Sachs O. (2013). For better or for worse? Empirical evidence of moral licensing in a behavioral energy conservation campaign. Energy Policy 57, 160–171. doi: 10.1016/j.enpol.2013.01.021

[ref90] Truelove H. B. Carrico A. R. Weber E. U. Raimi K. T. Vandenbergh M. P. (2014). Positive and negative spillover of pro-environmental behavior: an integrative review and theoretical framework. Glob. Environ. Change 29, 127–138. doi: 10.1016/j.gloenvcha.2014.09.005

[ref91] Truelove H. B. Nugent M. R. (2020). Straw wars: pro-environmental spillover following a guilt appeal. J. Environ. Psychol. 72:101521. doi: 10.1016/j.jenvp.2020.101521

[ref92] Truelove H. B. Yeung K. L. Carrico A. R. Gillis A. J. Raimi K. T. (2016). From plastic bottle recycling to policy support: an experimental test of pro-environmental spillover. J. Environ. Psychol. 46, 55–66. doi: 10.1016/j.jenvp.2016.03.005

[ref93] Tuk M. A. Prokopec S. Van den Bergh B. (2021). Do versus don't: the impact of framing on goal-level setting. J. Consum. Res. 47, 1003–1024. doi: 10.1093/jcr/ucaa050

[ref94] Van der Werff E. Steg L. Keizer K. (2013). It is a moral issue: the relationship between environmental self-identity, obligation-based intrinsic motivation and pro-environmental behaviour. Glob. Environ. Change 23, 1258–1265. doi: 10.1016/j.gloenvcha.2013.07.018

[ref95] Van der Werff E. Steg L. Keizer K. (2014a). Follow the signal: when past pro-environmental actions signal who you are. J. Environ. Psychol. 40, 273–282. doi: 10.1016/j.jenvp.2014.07.004

[ref96] Van der Werff E. Steg L. Keizer K. (2014b). I am what I am, by looking past the present: the influence of biospheric values and past behavior on environmental self-identity. Environ. Behav. 46, 626–657. doi: 10.1177/0013916512475209

[ref97] Varotto A. Spagnolli A. (2017). Psychological strategies to promote household recycling: a systematic review with meta-analysis of validated field interventions. J. Environ. Psychol. 51, 168–188. doi: 10.1016/j.jenvp.2017.03.011

[ref98] Wang C. Chu Z. Gu W. (2021). Participate or not: impact of information intervention on residents’ willingness of sorting municipal solid waste. J. Clean. Prod. 318:128591. doi: 10.1016/j.jclepro.2021.128591

[ref99] White K. MacDonnell R. Dahl D. W. (2011). It’s the mind-set that matters: the role of construal level and message framing in influencing consumer efficacy and conservation behaviors. J. Mark. Res. 48, 472–485. doi: 10.1509/jmkr.48.3.472

[ref100] Whitmarsh L. O’Neill S. (2010). Green identity, green living? The role of pro-environmental self-identity in determining consistency across diverse pro-environmental behaviours. J. Environ. Psychol. 30, 305–314. doi: 10.1016/j.jenvp.2010.01.003

[ref101] Williams L. A. DeSteno D. (2008). Pride and perseverance: the motivational role of pride. J. Pers. Soc. Psychol. 94, 1007–1017. doi: 10.1037/0022-3514.94.6.1007, PMID: 18505314

[ref102] Wolstenholme E. Poortinga W. Whitmarsh L. (2020). Two birds, one stone: the effectiveness of health and environmental messages to reduce meat consumption and encourage pro-environmental behavioral spillover. Front. Psychol. 11:577111. doi: 10.3389/fpsyg.2020.577111, PMID: 33117243 PMC7575709

[ref103] Wu Z. X. Guo T. T. Li B. K. (2021). Message framing’s role in encouraging idle item recycling. Asia Pac. J. Mark. Logist. 33, 1758–1775. doi: 10.1108/APJML-03-2020-0135

[ref104] Ye J. Yao Y. Li L. (2022). The more involved, the more willing to participate: an analysis of the internal mechanism of positive spillover effects of pro-environmental behaviors. J. Clean. Prod. 375:133959. doi: 10.1016/j.jclepro.2022.133959

[ref105] Yoon A. Jeong D. Chon J. Yoon J. H. (2019). A study of consumers’ intentions to participate in responsible tourism using message framing and appeals. Sustainability 11:865. doi: 10.3390/su11030865

[ref106] Zhang M. Zhang G. Y. Gursoy D. Fu X. R. (2018). Message framing and regulatory focus effects on destination image formation. Tour. Manag. 69, 397–407. doi: 10.1016/j.tourman.2018.06.024

